# Editorial: Insights in red blood cell physiology: 2021

**DOI:** 10.3389/fphys.2022.993287

**Published:** 2022-08-31

**Authors:** Lars Kaestner, Anna Bogdanova

**Affiliations:** ^1^ Theoretical Medicine and Biosciences, Saarland University, Homburg, Germany; ^2^ Experimental Physics, Saarland University, Saarbrücken, Germany; ^3^ Red Blood Cell Research Group, Institute of Veterinary Physiology, University of Zürich, Zürich, Switzerland

**Keywords:** band 3 protein, blood rheology, vesicular hemoglobin, erythropoietic protoporphyria, metabolomics, blood storage, pyruvate kinase deficiency, ApoE/LDLR double-deficient mouse

The topic “*Insights in Red Blood Cell Physiology 2021*” is dedicated to the recent advances and future perspectives in the field of Red Blood Cell Physiology with no focus on specific research area within the field. Since the particular contributions were unsolicited, the content of this topic is not an editor’s composition but reflect the submissions of the community.

To place the “Red Blood Cell Physiology” section in the context, we had a look into the Journal Citation Reports for 2021 (Journal Citation Reports, 2022). Within the Physiology category “Frontiers in Physiology” made it to rank 20 out of 81 journals and thus is placed within the first quartile of the journals - our congratulation! In haematology the competition is a bit tougher “Frontiers in Physiology” with the 2021 impact factor of 4.755 would range between rank 32 and 33 out of 79 journals, i.e., in the second quartile. This means “Frontiers in Physiology” is (naturally) more attractive in the field of physiology compared to haematology. Considering that “red blood cells” comprise only a subsection of haematology that is often combined with oncology, we like to state, that we are more than happy to have “Frontiers in Physiology” as a publication platform for red blood cell physiology.

In the Research Topic, biophysics is represented by the review article ‘*Organization and Dynamics of the Red Blood Cell Band 3 Anion Exchanger SLC4A1: Insights From Molecular Dynamics Simulations*’ by Kalli and Reithmeier, summarizing the current advances in our knowledge on the most abundant and versatile proteins in red blood cell membrane, anion exchanger 1 (AE1) or band 3 protein. During the past decades this protein was described as a key participant in bicarbonate transport, major anion transporter, a flippase, a docking site for deoxyhemoglobin, glycolytic proteins and a major structural element of the membrane cytoskeleton, a target for naturally occurring antibodies, that are tagging the cells for (Bamberg and Passow, 1992; Ortwein et al., 1994; Lutz, 2012; Chu et al., 2016). The review presented here, puts the focus on the function of band 3 protein through the lens of its structure and molecular dynamics pointing out the role of post-translational modifications in it. AE1 is one of the key structural elements of the cytoskeletal network in control of “vertical interactions” between the spectrin and transmembrane protein complexes that define membrane stability and contribute to red blood cell rheological properties. In their review “*Physical properties of blood and their relationship to clinical conditions*”, Alexy et al. extend the topic of major determinants of red blood cell rheological properties to the cytosolic elements, cell-cell interactions between red blood cells, the role of plasma in blood rheology, the factors influencing haemodynamics in pathology. Release of haemoglobin from the ageing stored red blood cells in the form of free molecules and as cargo of the released vesicles was investigated by Tzounakas et al. and is presented in the study “*Deciphering the Relationship Between Free and Vesicular Hemoglobin in Stored Red Blood Cell Units*”. This process also interferes with haemodynamics as it induces oxidative stress and limits NO bioavailability interfering with its vasorelaxant function. Progressive red blood cell membrane loss produces microcytes in patients with hereditary spherocytosis. However, other forms of rare hereditary red blood cell related diseases such as erythropoietic protoporphyria associated with deficiency in ferrochelatase that catalyzes insertion of iron into the haeme are also associated with microcytosis. In their work “*Microcytosis in Erythropoietic Protoporphyria*”, Graziadei et al. follow the association between the accumulation of protoporphyrin IX and reduced mean cell volume that exists in patients even in the absence of iron deficiency or anemia. They state that the mechanism behind remains unclear. However, we like to propose the involvement of photodynamic effects of protoporphyrin IX directly in the red blood cells (Kaestner et al., 2004).

Metabolomics and metabolism are clearly leading as hot-spots in the area of red blood cell biochemistry. Detailed analysis of metabolic disbalance, oxidative stress and hypoxia associated with pyruvate kinase deficiency is presented by Roy et al., in their contribution “*Red Blood Cell Metabolism in Pyruvate Kinase Deficient Patients*”. Due to the lack of gene editing technologies as routine clinical tool to treat this hereditary hemolytic anemic condition, alternative supportive therapeutic strategies will be based on the findings provided by such approaches as metabolomics. The power of the high-throughput metabolomics platform in defining the best conditions for blood storage and assessment of its quality is presented by Nemkov et al. In the study “*High-Throughput Metabolomics Plattform for the Rapid Data-Driven Development of Novel Additive Solutions for Blood Storage*”, the authors identify some strategies to refine the storage conditions, including reduced oxygenation, supplementation of antioxidants and substrates aiming and prolongation of storage time and improving the outcome of blood transfusions. The study “*Sex-Specific Differences of Adenosine Triphosphate Levels in Red Blood Cells Isolated From ApoE/LDLR Double-Deficient Mice*” by Alcicek et al. explores the impact of sexual dimorphism on ATP levels in red blood cells in a mouse model of atherosclerosis. Sex-specific alterations in the intra-erythrocytic ATP content, mechanical and morphological properties of red blood cells were found in an ApoE/LDLR double-deficient mouse model during the progression of atherosclerosis. These findings reveal the particular importance of the correct choice the animal model of disease, but also suggest that differential gender-specific approach may be required for effective therapy of atherosclerosis in humans.

This Research Topic is a snapshot in time showing some fascinating findings in several areas of red blood cell research, that will be followed up by the updated collection on further advances that will be brought by the year 2022 and beyond.
